# Sleep Architecture and Hypothalamic-Pituitary-Adrenal Activity in Paradoxical and Psychophysiological Insomnia

**DOI:** 10.32598/bcn.9.6.397

**Published:** 2018-11-01

**Authors:** Hiwa Mohammadi, Mohammad Rezaei, Seyed Mojtaba Amiri, Zohreh Rahimi, Kamran Mansouri, Habibolah Khazaie

**Affiliations:** 1. Sleep Disorders Research Center, Kermanshah University of Medical Sciences, Kermanshah, Iran.; 2. Department of Neurology, Faculty of Medicine, Kermanshah University of Medical Sciences, Kermanshah, Iran.; 3. Medical Biology Research Center, Kermanshah University of Medical Sciences, Kermanshah, Iran.

**Keywords:** Cortisol, Adrenocorticotropic Hormone (ACTH), Sleep disorder, Insomnia

## Abstract

**Introduction:**

There are controversial reports about association between sleep and Hypothalamic-Pituitary-Adrenal (HPA) activity. Studies have reported the influence of insomnia on HPA hormones. However, they usually ignored the heterogeneity of insomnia symptoms, so subtypes of the disorder have not been considered in the reports. The present study aimed to investigate the final and intermediate products of HPA system among a group of psychophysiological and paradoxical insomniac patients in comparison to a group of normal sleepers.

**Methods:**

We investigated the awakening serum level of Adrenocorticotropic Hormone (ACTH) and cortisol after one night Polysomnography (PSG) in 17 subjects with psychophysiological insomnia, 19 subjects with paradoxical insomnia and 17 subjects with normal sleep profile. Groups were matched for age and Body Mass Index (BMI). Serum levels of ACTH and cortisol were measured by Enzyme-Linked Immunosorbent Assay (ELISA) method.

**Results:**

Although, a tendency toward elevation of both ACTH and cortisol was observed among patients with paradoxical insomnia compared to both control and psychophysiological insomnia, the differences were not significant comparing three groups. According to regression analysis, higher Non-Rapid Eye Movement sleep (NREM) arousal and Pulse Transit Time (PTT) significantly predicted higher level of ACTH.

**Conclusion:**

These findings could suggest the personality traits hypothesis for paradoxical insomnia. Both cortical and subcortical arousal could lead to more HPA activity and higher ACTH level. Further studies are recommended to confirm the hypothesis.

## Highlights

- There is no association between insomnia and hypothalamic-pituitary-adrenal axis activity.
- Higher level of adrenocorticotropic hormone was predicted by higher non-rapid eye movement arousal state and pulse transit time.
- Personality traits may play a critical role in paradoxical insomnia.
- Both cortical and subcortical arousal state may induce more hypothalamic-pituitary-adrenal activity.

## Plain Language Summary

Sleep has a critical role in endocrine system. Insomnia is an important sleep disorder that could disrupt body homeostasis and health. This disorder may result from endocrine problems. In this study, the activity of stress systems was compared between normal sleepers and people with insomnia. We hypothesized that insomnia disorder may lead to malfunction of stress systems in human. The level of awakening cortisol and Adrenocorticotropic Hormone (ACTH) were measured and a wide range of sleep physiological signals were recorded during night sleep. A group of people with insomnia and a group of normal sleepers were studied and the obtained results were compared. In addition, associations between sleep physiological signals and hormones were analyzed. In contrast to acceptable clinical attitude, the present findings suggest that insomnia may have not significant consequences on stress systems, including stress hormones and autonomic nervous system. Higher number of arousal states during slow wave stage as a more deep stage of sleep may disrupt the refreshing effect of sleep on brain mechanisms related to hypothalamus-pituitary-adrenal modulation. Total Non-Rapid Eye Movement (NREM) arousal states could be considered as a good index of sleep fragmentation during slow wave sleep, the sleep stages that have a more recovery outcomes on brain physiology.

## 1. Introduction

Sleep is an important body homeostatic behavior that plays a critical role in emotional and cognitive functions (Koren et al., 2011). Poor sleep quality could also disrupt the endocrine system (Ioja, Weir, & Rennert, 2012; Mullington, Simpson, Meier-Ewert, & Haack, 2010; Potter et al., 2016). In particular, sleep duration and quality could alter biological systems responses to stress (Meerlo, Sgoifo, & Suchecki, 2007). Biological rhythms that regulate two main stress systems, i.e. Hypothalamus-Pituitary-Adrenal (HPA) and Autonomic Nervous System (ANS) are controlled by Suprachiasmatic Nucleus (SCN) of hypothalamus.

SCN as a circadian pacemaker is also responsible for modulation of sleep-wake cycle (Hastings, Reddy, & Maywood, 2003). Previous studies have emphasized that the circadian mechanisms which control the sleep, also directly modulate ANS (Leproult & Van Cauter, 2010). Sleep disturbances change the autonomic activity and increase heart rate and blood pressure (Zhong et al., 2005). In addition, brain centers that control sleep-wake cycle and stress-responsive biological systems are closely interrelated. Therefore, sleep duration may change the patterns of stress systems (Edwards, Evans, Hucklebridge, & Clow, 2001).

Insomnia is the most prevalent sleep disorder that affects 10% of adult population (Morin & Benca, 2012; Ohayon & Reynolds, 2009). It is defined as difficulties in initiating and maintaining sleep, or early-morning awakening with inability to return to sleep. To call it insomnia, it should occur at least three times per week, and produce clinically significant impairment in social, occupational or other aspects of subject’s functions (American Psychiatric Association, 2013). Despite the high prevalence of insomnia and its adverse health and economic outcomes, the biological mechanisms of bidirectional interaction between stress systems and sleep have not been fully understood.

Although there is a physiological link between neural representation of sleep-wake cycle and stress-responsive systems, inconsistent results have been reported about association between sleep architectures and cortisol level, the final product of HPA (Elder, Wetherell, Barclay, & Ellis, 2014). Association between sleep duration and awakening cortisol level has been already reported. Based on a study conducted on 58 normal adults on the association among sleep, stress and cortisol awakening response, lower subjective total sleep time is significantly related to lower cortisol levels at awakening (Vargas & Lopez-Duran, 2014).

Similarly, reduced cortisol level after 24 h sleep deprivation has been reported (Arnal et al., 2016). In contrast, increased serum cortisol level has been reported among people with insomnia in many previous studies, too (Floam et al., 2015; Meerlo et al., 2007; Rodenbeck & Hajak, 2001; Rodenbeck, Huether, Rüther, & Hajak, 2002).

Some studies failed to find any association between sleep duration and cortisol level. Federenko et al. (2004) reported a relation between awakening time and cortisol level but no association between sleep duration and cortisol. Moreover, some studies did not indicate any association between sleep and HPA axis activity at all (Lattova et al., 2011; Varkevisser, Van Dongen, & Kerkhof, 2005). These different results that ranged from positive or negative association to no relation between sleep and HPA activity call for more investigation in this area. Further research by well-control equipment for objective analysis of sleep architectures would be helpful for explaining these discrepancies in results.

Broad controversies over the association of sleep with HPA activity indicate that this association is not a static unidirectional relation but a dynamic one modulated by different aspects of sleep characteristics. A recent study reports that sleep deprivation associates with higher morning cortisol levels, whereas sleep misalignment leads to lower morning cortisol levels (Wright et al., 2015). In addition, research on the association between sleep and HPA activity among patients with insomnia has revealed inconsistent results. Although some studies report an increased serum cortisol level in people with insomnia (Floam et al., 2015; Meerlo et al., 2007; Rodenbeck & Hajak, 2001; Rodenbeck et al., 2002), some others did not find any significant association (Dahlgren, Kecklund, Theorell, & Akerstedt, 2009; Eek, Karlson, Garde, Hansen, & Orbaek, 2012; Hsiao et al., 2013; Riemann et al., 2002).

Using subjective self-reported questionnaires or objective laboratory-based equipment may lead to different findings. In some studies, self-reported insomnia symptoms were associated with lower waking cortisol. For example, Backhaus, Junghanns and Hohagen (2004) reported a significantly lower cortisol after awakening in patients with primary insomnia compared to control. They reported negative correlation between subjective sleep quality measured by Pittsburgh Sleep Quality Index (PSQI) and salivary awakening cortisol. Moreover, it has been reported that sleep problems during the past month were associated with low morning and evening salivary cortisol level (Hansen et al., 2012). On the other hand, significant association between objective sleep characteristics (measured by actigraphy and diurnal cortisol patterns) has been reported (Castro Diehl et al., 2015).

Different characteristics of sleep and various methods for investigating sleep architectures may explain these controversial reports. Previous studies have usually considered sleep duration and quality for investigating the association between sleep and HPA. Nevertheless, there are important sleep EEG characteristics and Polysomnography (PSG) data on related physiological system that may provide more information on the relationship between HPA and sleep homeostasis. Our knowledge about the relation of sleep architecture and sleep EEG structures with HPA activity is limited. Based on another recent study, up-regulation of cortisol is associated with wake after sleep onset using actigraphy and diary based sleep monitoring (Floam et al., 2015). According to this finding, sleep fragmentation may be a more potent factor influencing HPA activity compared to total sleep time and sleep efficiency. Therefore, PSG is a more suitable method for detection of the breakup events during sleep and may appropriately reveal interactions between sleep and HPA activity.

Another important gap in studies of insomnia is ignoring the subtypes of the disorder. Inconsistent findings about association between sleep and HPA activity may simply because of overlooking the symptomatically heterogeneous nature of insomnia (Floam et al., 2015). Previous studies reported the HPA data on insomnia on the whole, but studies on the subtypes of the disorder such as paradoxical insomnia were rare. Paradoxical insomnia is a sleep state misperception condition and defined as an insomnia without objective findings. This subjective but not objective insomnia is a subtype of primary insomnia, with prevalence ranging between 9.2% and 50% in patients with insomnia (American Academy of Sleep Medicine, 2002).

Patients with paradoxical insomnia report little to no sleep at all over long periods of time, but objective sleep findings such as PSG reveal near-normal sleep patterns in these people (Edinger & Krystal, 2003). Additionally, they do not display the level of daytime sleepiness that experienced after sleep deprivation (American Academy of Sleep Medicine, 2002). Considering the PSG and actigraphy results that paradoxically are similar to normal sleeper in spite of insomnia complaint, the HPA activity would be a discriminative index for distinguishing paradoxical insomnia from normal sleeping and psycho-physiological insomnia.

An important etiological hypothesis suggests the role of personality traits in paradoxical insomnia (Dorsey & Bootzin, 1997; Vanable, Aikens, Tadimeti, Caruana-Montaldo, & Mendelson, 2000). However, there is no consensus over the possible causal role of these personality traits in paradoxical insomnia (Dorsey & Bootzin, 1997). It has been suggested that these traits may increase the anxiety level and subsequent misperception of sleep (Harvey & Tang, 2012). Therefore, the study of biological systems responsible for stress could test this hypothesis and reveal the associations between sleep misperception and stress system.

In the present study, we aimed to investigate the serum levels of final and intermediate products of HPA system among a group of patients with psychophysiological and paradoxical insomnia in comparison to a group of normal sleepers. In addition, associations between HPA activity and objective sleep architectures as well as autonomic activity were investigated.

## 2. Methods

### 2.1. Study participants

Thirty-six consecutive patients with insomnia complaint who were referred to Sleep Disorders Research Center (SDRC) of Kermanshah University of Medical Sciences (KUMS) from April 2014 to October 2016 were recruited to participate in the study. They comprised 21 (58.30%) females and 15 (41.70%) males aged 14 to 62 years (Mean±SD age: 41±12.20 y). Twenty-one subjects with normal sleep consisted of 4 (19%) females and 17 (81%) males aged 26 to 59 years (Mean±SD age: 40.70±10 y) were invited from Kermanshah Province as the control group. They were matched with insomnia groups for age and BMI. The study was approved by the Ethics Committee of KUMS and all participants completed detailed written informed consent.

The exclusion criteria were having chronic medical disorders, other sleep problems, any substances use, neurological disorders, psychiatric conditions, respiratory problems or cardiovascular disorders. Participants stopped using medications such as benzodiazepines that may affect sleep characteristics 2 weeks before data collection. Other sleep conditions such as hypersomnia, parasomnias, circadian sleep-wake disorders, and restless legs syndrome identified by physician’s examinations, and sleep breathing and periodic limb movement disorders identified by PSG were also criteria for exclusion from the study.

### 2.2. Insomnia diagnosis and subjective sleep investigation

The insomnia was diagnosed by sleep clinician according to ICSD2 (the International Classification of Sleep Disorders) based clinical interview (American Academy of Sleep Medicine, 2002). At the first step, all participants with insomnia and normal participants were clinically interviewed by an experienced psychiatrist who had fellowship in sleep medicine according to ICSD2, second edition (American Academy of Sleep Medicine, 2002) criteria. Participants in insomnia group were referred to the second step for PSG investigation if they were diagnosed as insomnia according to ICSD2. In addition, the volunteers in normal control group were also interviewed and those who had not any sleep disorder were selected for the second step of the study.

Subjective diagnostic criteria for insomnia included 1. Subjective complaining of insomnia characterized by difficulties in initiating and or maintaining sleep; 2. Having insomnia at least three nights a week for more than six months; 3. Complaining of at least one daytime consequence attributed to insomnia; 4. Having distress or significant difficulties in social and/or occupational functioning; and 5. Having subjective Sleep Efficiency (SE) below 85% in their two week sleep diary prior to PSG recordings. Four participants in the control group were excluded from the study due to symptoms of Obstructive Sleep Apnea (OSA) and did not go to the next step according to the first interview. In the next step, participants were invited for performing a whole night PSG procedure. The inclusion and exclusion criteria for both patients and control groups were confirmed by PSG results.

Subjective sleep quality over the past month was measured by PSQI in a self-reported manner (Buysse, Reynolds, Monk, Berman, & Kupfer, 1989). The questionnaire consists of 19 individual items assessing 7 components of sleep quality, sleep latency, Total Sleep Time (TST), Sleep Efficiency (SE), sleep disturbances, use of sleeping medication, and daytime dysfunction. We considered subjective TST, SE and sleep latency for analysis in the present study.

### 2.3. PSG procedure

To record the sleep physiological parameters, the participants underwent an overnight polysomnography (SOMNO-screen plus®, Somnomedics, Germany). They were invited to sleep at the laboratory of SDRC and an appointment was scheduled according to the participant’s desire. They were advised not to take any snooze and sleep during the day before the appointment. In addition they should avoid consuming coffee, tea, heavy diet and cigarette as well. They should arrive the laboratory at 9 PM.

Then, the participants completed the demographic and PSQI questioners. PSG procedure was explained for the participants and their questions were answered. The PSG room was standardized for any noise and visual stimulus based on international standard (American Academy of Sleep Medicine, 2002). Recording of PSG started based on the subject’s usual sleeping habits, and each patient’ sleep was recorded for a minimum of 7 hours.

AASM guideline (2002) was considered for the measurement of PSG. Electroencephalogram was recorded using frontal, central and occipital leads according to 10–20 system. In addition, Electrooculogram (EOG), Electromyogram (EMG), oximetry, abdominal and thoracic respiratory effort (induction plethysmography), and body position were recorded. Oronasal thermocouples and nasal pressure transducers were used for monitoring the respiration. Continuous pulse oximetry was also monitored and thoracoabdominal movements were monitored by piezoelectric strain gauges.

Sleep latency of less than 15 minutes, SE of more than 85% and TST of greater than 7 hours were considered as objective normal sleep pattern for normal control group. The presence of marked discrepancies between subjective and objective sleep measures (i.e. a difference of one hour or more for TST, or a difference of at least 15% between subjective and objective measures of sleep efficiency), an objective TST of more than 6 hour and 30 min, and a SE greater than 85% on nocturnal PSG were considered as criteria for diagnosing of paradoxical insomnia (Edinger et al., 2004).

Finally, the patients with subjective insomnia diagnosis that failed to reach the above mentioned criteria and did not have other sleep conditions considered as having psychophysiological insomnia. TST, SE, sleep latency, Non-Rapid Eye Movement (NREM) sleep stages, total REM percent, total REM arousal state, total NREM arousal state, wake index, Wake After Sleep Onset (WASO) and Pulse Transit Time (PTT) were calculated and considered for further analysis. PTT is a time needed for receiving a pulse wave from heart to the finger. It has been considered as a blood pressure marker and related to ANS activity (Hey & Sghir, 2011).

### 2.4. Biochemical analysis

Five milliliters of venous blood was collected at 8 AM after PSG recording from all the participants. Serum was separated by centrifuge and stored at −20°C for the biochemical analyses according to standard protocols.

Serum levels of cortisol (Code 3625-300; Monobind Inc.) and ACTH (REF 7023, BIOMERICA) were measured by enzyme-linked immunosorbent assay kits. Awareness Technology STAT FAX 2100 Microplate Reader (Awareness Technology, USA) was used for reading the level of absorbance. Hormone levels were calibrated to the standard calibration curve of each hormone according to the kits. The obtained data were presented as pg/mL for ACTH and μg/dL for cortisol.

### 2.5. Statistical analysis

The obtained data from psychophysiological insomnia, paradoxical insomnia and control groups were analyzed by Chi-square and ANCOVA tests. Significant differences between groups were detected by post hoc Tukey multiple comparisons. The levels of hormones and sleep characteristics were compared between three groups and the age, gender and BMI were used as covariates. The relation between PSG sleep characteristics and biochemical parameters was evaluated by multiple linear regression analysis according to covariates of age, gender, BMI and group. All model assumptions were evaluated by residual analysis. SPSS (SPSS, Inc., Chicago, IL) version 16.0 was used for performing statistical analyses.

## 3. Results

### 3.1. Demographic findings

We recruited 57 subjects with the Mean±SD age of 41.52±11.30 years. Four participants in the control group were excluded from the study due to OSA diagnosis in the first psychiatric interview. Finally, 53 subjects with the Mean±SD age of 40.92±11.50 years were included in the study. In the normal control group, 17 participants completed the study. According to PSG criteria, patients were divided to 19 subjects including 13 (68.40%) females with paradoxical insomnia (Age range: 32–53 years; Mean±SD age: 43.20±6.40) and 17 subjects including 8 (47.10%) females with psychophysiological insomnia (Age range:14–62 years; Mean±SD age: 38.40±16.30 years) (Table 1).

**Table 1:** Comparison of demographic characteristics between normal sleepers and groups with insomnia

**Variables**		**Normal (n=17)**	**Paradoxical Insomnia (n=19)**	**Psychophysiological Insomnia (n=17)**	**P**
Age^†^, y		40.76±10.1^a^	43.26±6.45^a^	38.47±16.36^a^	0.466^c^
Sex	Female	4(23%)	13(68.4%)	8(25.0%)	0.027^b^
	Male	13(76.5%)	6(31.6%)	9(52.90%)	
BMI^†^, kg/m^2^		26.57±3.82^a^	26.55±3.8^a^	26.97±6.54^a^	0.961^c^

†: Mean±SD. Data compared by

c ANOVA and

b Chi-Square test. Means with the same superscript letters within a row are not significantly different (P>0.05). Abbreviations: Paradoxical Insomnia (Para-Insomnia); Psychophysiological Insomnia (Psych-insomnia).

Demographic characteristics of participants are presented in Table 1. Three groups were age- and BMI-matched. However, there are significantly higher numbers of females in paradoxical and psychophysiological insomnia groups. We considered the sex as a covariant in all analyses.

### 3.2. Objective and subjective sleep characteristics

We considered subjective TST, SE, and sleep latency obtained from PSQI for study analyses. In addition, PSG results including TST, SE, sleep latency, sleep efficiency, NREM sleep stages, total REM percent, total REM arousal state, total NREM arousal state, wake index, WASO, and PTT were considered for objective analyses. We adjusted the sex, age and BMI in all analyses. According to PSQI results, TST, SE, and sleep latency were significantly different between three groups (P<0.01). Post hoc analysis by Tukey test, indicated that subjective TST was significantly low in paradoxical insomnia group compared to normal sleepers (P<0.001) and psychophysiological insomnia (P=0.001). Also, in paradoxical insomnia group the subjective sleep latency was significantly higher than normal sleepers (P<0.001) and psychophysiological insomnia (P<0.001). In addition, subjective sleep efficiency in this group was lower than both normal sleepers (P<0.001) and psychophysiological insomnia group (P=0.002) (Table 2).

**Table 2:** Comparison of sleep structures demographic characteristics between normal sleepers and groups with insomnia

**Sleep Characteristics**	**Normal (n=17)**	**Paradoxical Insomnia (n=19)**	**Psychophysiological Insomnia (n=17)**	**P**
PSQI total sleep time^†^ (h)	5.57±1.93^a^	2.25±2.46^b^	4.60±2.73^a^	<0.01
PSQI sleep latency (h)	0.79(0.57)^a^	3.01(1.24)^b^	1.39(1.32)^a^	<0.01
PSQI sleep efficiency	74.78(25.96)^a^	28.88(20.49)^b^	58.28(32.66)^a^	0.01
PSG total sleep time	7.11(0.44)^a^	6.82(0.69)^ab^	6.02(1.58)^b^	0.01
PSG sleep latency	7.84(6.60)^a^	9.13 (6.58)^a^	15.30(26.40)^a^	0.07
PSG sleep efficiency	92.79(4.32)^a^	88.04(7.28)^a^	77.25(19.94)^b^	<0.01
N1 stage (%)	32.44(16.39)^a^	32.56(16.54)^a^	32.60(13.51)^a^	0.41
N2 stage (%)	21.67(9.19)^a^	20.21(10.27)^a^	16.28(9.33)^a^	0.32
N3 stage (%)	30.65(18.39)^a^	23.94(15.12)^a^	19.17(16.82)^a^	0.15
REM (%)	8.01(8.30)^a^	11.32(8.86)^a^	9.13(9.79)^a^	0.81
Total REM arousal state	20.50(11.05)^a^	23.86(12.62)^a^	24.31(18.28)^a^	0.93
Total NREM arousal state	24.76(9.02)^a^	24.74(6.26)^a^	22.99(5.43)^a^	0.69
PSG wake index	2.02(0.68)^a^	3.54(1.92)^a^	8.92(10.81)^b^	<0.01
WASO	14.53(4.68)^a^	23.36(11.94)^a^	40.58(28.32)^b^	<0.01
Maximum PTT	360.93(22.20)	356.16(24.97)	361.71(19.94)	0.73
Minimum PTT	262.97(10.02)	249.89(15.63)	266.18(28.92)	0.04
Average PTT	305.93(10.78)	299.79(17.91)	308.24(14.72)	0.23

†: Mean±SD. Statistical analysis for the equality of the mean values among the three groups was evaluated using ANCOVA adjusted by sex, age, and BMI (P<0.05). Means with the same superscript letters within a row were not significantly different (P>0.05). Abbreviations: Paradoxical Insomnia (Para-Insomnia); Psychophysiological Insomnia (Psych-insomnia); Pittsburgh Sleep Quality Index (PSQI); Polysomnography (PSG); Rapid Eye Movements (REM); Non-Rapid Eye Movement (NREM); Wake After Sleep Onset (WASO); and Pulse Transit Time (PTT).

Different results were obtained for objective sleep characteristics investigated by PSG. According to Tukey post hoc analysis test, psychophysiological insomnia presented significantly lower objective TST compared to normal sleepers (P=0.017) and lower but non-significant objective TST compared to paradoxical insomnia group (P=0.073).

Similarly, psychophysiological insomnia group had significantly lower sleep efficiency compared to normal sleepers (P=0.004) and paradoxical insomnia group (P=0.042) (Table 2). Although, objective sleep latency was not significantly different comparing three groups, but it was considerably high among patients with psycho-physiological insomnia compared to paradoxical insomnia and control groups (P=0.071). The PSG wake index was significantly higher among psychophysiological insomnia group compared to normal sleepers (P=0.014) and paradoxical insomnia groups (P=0.04). In addition, WASO was significantly higher among psychophysiological insomnia group compared to normal control (P=0.001) and paradoxical insomnia group (P=0.02) (Table 2).

**Figure 1:**
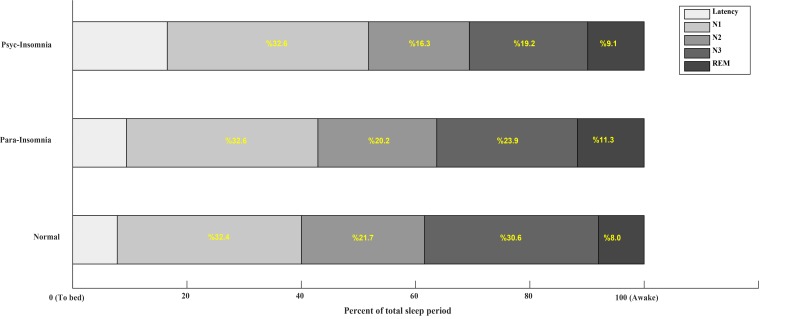
PSG sleep architecture among normal sleepers and two subtypes of insomnia Abbreviations: Paradoxical Insomnia (Para-Insomnia); Psychophysiological Insomnia (Psych-insomnia); stage 1 of non-rapid eye movement (N1); stage 2 of non-rapid eye movement (N2); stage 3 of non-rapid eye movement (N3); and Rapid Eyes Movement (REM).

Maximum, minimum and average PTT was lower in paradoxical insomnia group but the difference between groups was only significant in minimum PTT (P=0.047). According to the post hoc analysis, the difference of minimum PTT between paradoxical insomnia and normal sleepers was not significant (P=0.17). Similarly, there was no significant difference between paradoxical and psychophysiological insomniac groups in minimum PTT (P=0.05) (Table 2). A schematic representation of PSG sleep architecture in three studied groups is shown in Figure 1. According to the Figure, N3 stage decreased and REM stage increased in paradoxical and psycho-physiological insomniac groups compared to normal control group (Figure 1).

### 3.3. Biochemical findings

All biochemical analyses were compared between groups by ANCOVA after adjustment of sex, BMI, and age covariates. According to the results, there was no significant difference between normal sleepers and two subtypes of insomnia regarding the biochemical parameters (Table 3).

**Table 3:** Comparison of biochemical parameters between normal sleepers and groups with insomnia

**Hormones**	**Normal (n=17)**	**Paradoxical Insomnia (n=19)**	**Psychophysiological Insomnia (n=17)**	**P**
ACTH	25.08(17.94)^a^	28.28(13.82)^a^	21.94(12.39)^a^	0.40
Cortisol	134.09(51.11)^a^	153.86(62.87)^a^	133.40(39.32)^a^	0.45

Statistical analysis for the equality of the mean values among the three groups was evaluated using ANCOVA adjusted by sex, age, and BMI (P<0.05). Means with the same superscript letters within a row were not significantly different (P>0.05).

Association between PSG sleep structures and biochemical parameters was analyzed by linear regression model adjusted by sex, age, and BMI. According to the results, total NREM arousal state and maximum PTT had a significant effect on ACTH level (Coefficient=0.74; P=0.03). (Table 4). None of the PSG sleep characteristics influenced the cortisol level.

**Table 4:** Multiple analysis of variables associated with ACTH using the linear regression model adjusted by sex, age, and BMI

**Variables**		**Coefficient**	**95% CI**		**P**

			**Lower**	**Upper**	
ACTH	Total non-REM arousal state	0.673	0.029	1.266	0.031
	Maximum pulse transit time	0.214	0.006	0.404	0.043

## 4. Discussion

Insomnia is the most prevalent sleep condition that could disrupt body homeostasis and affect many physiological systems. Control of circadian rhythm and stress system in the brain overlap anatomically and physiologically. Both sleep circadian rhythm and stress-responsive biological systems are affected and regulated by SCN in hypothalamus (Hastings et al., 2003). According to previous studies, sleep-wake cycle and stress-responsive biological systems are closely interrelated, so that sleep duration may change the patterns of stress systems (Edwards et al., 2001). Therefore, the effect of insomnia on HPA has been suggested and investigated in methodologically different studies with inconsistent results.

Ignoring the subtypes of insomnia and various sleep characteristics in previous studies motivated us to investigate the ACTH and cortisol, as intermediate and final products of HPA respectively, in two main subtypes of primary insomnia, i.e. psychophysiological and paradoxical insomnia. To the best of our knowledge, the present study is the first investigation about HPA activity among people with paradoxical insomnia.

Results of the present study did not indicate any significant differences between normal sleepers and both subtypes of insomnia with respect to ACTH and cortisol levels. However, both cortisol and ACTH levels among psychophysiological insomnia were non-significantly lower than paradoxical insomniac and normal sleeper groups. Previous studies indicate that shorter sleep durations lead to lower levels of waking cortisol in the next day (Van Lenten & Doane, 2016; Vargas & Lopez-Duran, 2014). In addition, subjectively-defined insomnia has been associated with normal (Rodenbeck et al., 2003; Rodenbeck et al., 2002; Vgontzas et al., 2001; Vgontzas et al., 1998) or decreased morning awakening cortisol levels (Backhaus et al., 2004). In contrast, some studies report up-regulation of cortisol among insomnia patients compared to controls (Floam et al., 2015).

According to PSG data, people with psychophysiological insomnia have significantly lower objective sleep duration and sleep efficiency compared to paradoxical insomnia and normal sleepers. Thus, lower awakening ACTH and cortisol levels are obtained, although the difference is not significant that may be due to low sample size. On the other hand, both ACTH and cortisol levels were non-significantly higher among people with paradoxical insomnia compared to normal group and patients with psychophysiological insomnia.

According to the present results, the psychophysiological subtype of insomnia does not have any significant effect on cortisol level as a final product of HPA. These findings suggest that psychophysiological insomnia may not have significant consequences on stress system. However, the condition for paradoxical insomnia might be completely different. Paradoxical insomnia is a sleep state misperception condition (American Academy of Sleep Medicine, 2005). Considerable mismatch between objective and subjective sleep architecture has been introduced as core diagnostic criteria for paradoxical insomnia (Edinger & Krystal, 2003).

PSQI subjective total sleep time and sleep efficiency was significantly lower among paradoxical insomnia compared to normal and even psychophysiological insomnia. Nevertheless, the PSG total sleep time and sleep efficiency were similar to normal group. Therefore, PSG results in the group with paradoxical insomnia were similar to normal sleeper in spite of insomnia complaint. Future studies with larger sample size on the effect of paradoxical insomnia on HPA activity may reveal the role of stress system in this sleep misconception condition. The role of personality traits in the etiology of paradoxical insomnia has been also suggested (Dorsey & Bootzin, 1997; Vanable et al., 2000). These traits may lead to higher anxiety level and subsequent misperception of sleep (Harvey & Tang, 2012). The higher anxiety level may be expressed by higher ACTH and cortisol levels, although the results of present study did not indicate a significant effect, which may be due to low sample size.

After controlling of age, gender, and BMI effects in studied groups, one of PSG variables that could significantly affect the serum ACTH level was total NREM arousal state. This index could be considered as an electrocortical arousal and sleep fragmentation sign. According to a previous study that used the subjective measure, higher frequency of awakening during night was directly correlated with awakening salivary cortisol level (Backhaus et al., 2004). Total NREM arousal state could be considered as a good index of sleep fragmentation during slow wave sleep, the sleep stages that have more recovery outcomes on brain physiology. We suggest that higher number of arousal states during slow wave sleep may disrupt the refreshing effect of sleep on brain mechanisms related to HPA modulation.

Another variable that predict the ACTH level is maximum PTT. PTT is considered as a marker for blood pressure and could be related to ANS activity. It has also been introduced as an appropriate measurement for stress (Hey & Sghir, 2011; Hey et al., 2009). Therefore, the association between maximum PTT and serum ACTH level may be due to similar neurophysiological mechanisms responsible for both PTT and ACTH and also the top-down higher control mechanisms modulating both HPA and PTT simultaneously.

The minimum PTT was significantly different between groups and was low among patients with paradoxical insomnia. It has been suggested that EEG arousal index could not indicate subcortical arousal events that modulate respiratory and blood pressure conditions. Therefore, PTT is a more suitable index for evaluating subcortical arousal that exerts vegetative and physiological consequences on stress system (Katz, Lutz, Black, & Marcus, 2003). Considering PTT as a subcortical arousal may open a new window for investigation of insomnia. Previous studies have considered insomnia as a subjective judgment of short duration and less efficient sleep. By advanced technologies such as actigraphy and PSG, objective measurement of sleep duration and efficiency have been used in sleep studies.

The EEG monitoring of sleep records surface electrophysiological activity of cortex, but neurophysiological events such as Reticular Activating System (RAS) in the brain stem that contain important arousal modulator is ignored. Thus considering PTT as an index for subcortical arousal state may provide important information for understanding the biological bases of insomnia.

People who suffer from insomnia do not present significant differences compared to controls in awakening serum ACTH and cortisol levels. However, a tendency toward the elevation of both ACTH and cortisol levels is observed among patients with paradoxical insomnia compared to both control and psychophysiological insomniac groups. Further studies with large sample size are recommended in this regard. Higher level of ACTH is significantly predicted by higher NREM arousal state and PTT. Therefore, both cortical and subcortical arousal state may lead to more HPA activity and higher ACTH level.

Some limitations should be taken into account when interpreting our results. The objective sleep duration in this study was based on one night PSG monitoring that may not represent habitual sleep duration and may be affected by first-night effects. In addition, we only measured awakening serum cortisol levels and could not measure cortisol awakening response.

## Ethical Considerations

### Compliance with ethical guidelines

The study was approved by the Ethical Committee of Kermanshah University of Medical Sciences (Ref: IR.KUMS.REC.1395.265) and written consent forms were achieved from all participants.
